# Ten years on: Is dental general anaesthesia in childhood a risk factor for caries and anxiety?

**DOI:** 10.1038/sj.bdj.2017.175

**Published:** 2017-02-24

**Authors:** S. Haworth, T. Dudding, A. Waylen, S. J. Thomas, N. J. Timpson

**Affiliations:** 1 Medical Research Council Integrative Epidemiology Unit (IEU) at the University of Bristol, Bristol; 2 School of Oral and Dental Sciences, University of Bristol Dental Hospital, Bristol; 3 School of Social and Community Medicine, University of Bristol, Bristol

## Abstract

Objectives To identify whether dental general anaesthesia (DGA) status is informative in assessing risk of caries or dental anxiety by (a) describing long-term oral health and dental anxiety for people who underwent DGA in childhood and (b) testing whether DGA status in childhood is associated with incident future dental caries or anxiety independently of preconceived risk factors.

Design Analysis of prospectively obtained data.

Setting An established population based cohort in the UK, the Avon Longitudinal Study of Parents and Children.

Participants and methods In total 1,695 participants with dental data in childhood and adolescence were included in analysis. DGA status by age 7 and oral health measures at age 17 were identified from questionnaire data.

Main outcome measures Filled or extracted permanent teeth at age 17, Corah Dental Anxiety Scale.

Results One hundred and twenty-eight (7.6%) participants underwent DGA in childhood. Individuals who underwent DGA had higher measures of filled or extracted permanent teeth in adolescence (0.36 more affected teeth in fully-adjusted model [95% confidence interval: 0.27, 0.55; P <0.001]).

Conclusions DGA in childhood predicts burden of treated caries in adolescence, independently of other risk factors. DGA status may be a clinically useful adjunct in identifying young people at high risk of further disease.

## Introduction

Dental caries in childhood remains a major public health problem in the UK, affecting 49% of children by age 5.^1^ Predicting risk of dental caries in childhood is therefore of major interest to families and clinicians. A number of studies have been conducted to identify predictors of caries risk in childhood, resulting in a range of approaches and tools. These have recently been reviewed by Divaris, who highlighted the limited utility and weak evidence supporting a number of existing systems.^2^

Existing systems for caries risk assessment have consistently highlighted socioeconomic status (SES) and previous dental caries as predictive of concurrent dental caries,^3^,^4^,^5^,^6^,^7^ however these measures have limitations in clinical practice. Assessment of socioeconomic status is subjective, and detailed questions about home circumstances may be perceived as intrusive. Previous dental caries may not be identified on clinical examination, for example if carious teeth in the primary dentition have been extracted or exfoliated.

An additional way to assess risk could be based on dental treatment under general anaesthesia (DGA) status. Tooth extraction under DGA is now the leading cause of hospital admission for children aged 5–9 years old,^8^ with trends for increasing admissions and younger age at admission.^9^,^10^,^11^

DGA is carried out where other methods of pain and anxiety control have not been successful or are deemed impractical. This may be due to a severe or early presentation of dental caries, or anxiety regarding dental procedures. It is possible that children who undergo DGA have high exposure to environmental or behavioural risk factors, such as prolonged or inappropriate bottle feeding, poor diet or poor oral hygiene practices. DGA status may act as a proxy for these risk factors, but is not currently used in any of the major risk assessment tools.^3^,^4^,^5^,^6^,^7^ It is also possible that children who undergo DGA have already been exposed to primers for dental anxiety or avoidance. We hypothesise that DGA status could assist long term caries risk assessment by proxying caries risk factors, and that DGA in childhood could identify people at risk of long term dental anxiety or avoidance. If so, DGA status could be used to identify individuals that require closer follow-up, more aggressive preventative measures or modified approaches to reduce dental anxiety.

Existing published evidence does not allow us to test these hypotheses. DGA status may already be used in risk assessment by some clinicians but there is neither published evidence supporting this nor evidence for how DGA status compares to commonly used risk assessment criteria. More generally there is a lack of published evidence about the long term oral health of people after DGA. In one dental teaching hospital in the UK, 34% of children required additional dental treatment during the six years after DGA, while other studies report repeat DGA rates between 4.2% and 17.0%^12^,^13^ in this population. These high re-attendance rates imply high levels of new incident disease. Other studies highlight that parents of this high-risk group are likely to lack oral health knowledge and that there are low levels of ongoing oral health support for children who have had DGA.^14^,^15^,^16^,^17^

Existing studies show little difference in dental anxiety between DGA and control groups but are limited by very short follow up times (up to 4 weeks).^16^,^17^ It is currently unknown whether DGA status is associated with measures of dental anxiety in the longer term.

This study aimed to (a) describe long-term oral health and dental anxiety for people who underwent DGA in childhood, (b) test whether DGA status in childhood is associated with measures of dental caries or dental anxiety in adolescence and (c) establish whether these associations are independent of preconceived risk factors.

## Participants and methods

Analysis of prospectively obtained data was carried out within an established birth cohort, the Avon Longitudinal Study of Parents and Children (ALSPAC).

### Participants

ALSPAC is a prospective cohort that initially recruited 14,541 pregnant women resident in Avon, UK with expected dates of delivery between 1st April 1991 and 31st December 1992.^18^ Including two later rounds of recruitment there were 15,445 eligible children enrolled in the study. Approval for this analysis was obtained from the ALSPAC Ethics and Law Committee, which is registered as an institutional review board. Parents gave written informed consent for the participation of their children in the study. Younger children gave assent, while older children gave consent for participation. Please note that the study website contains details of all the data that are available through a fully searchable data dictionary (http://www.bris.ac.uk/alspac/researchers/data-access/data-dictionary/).

**Figure 1:**
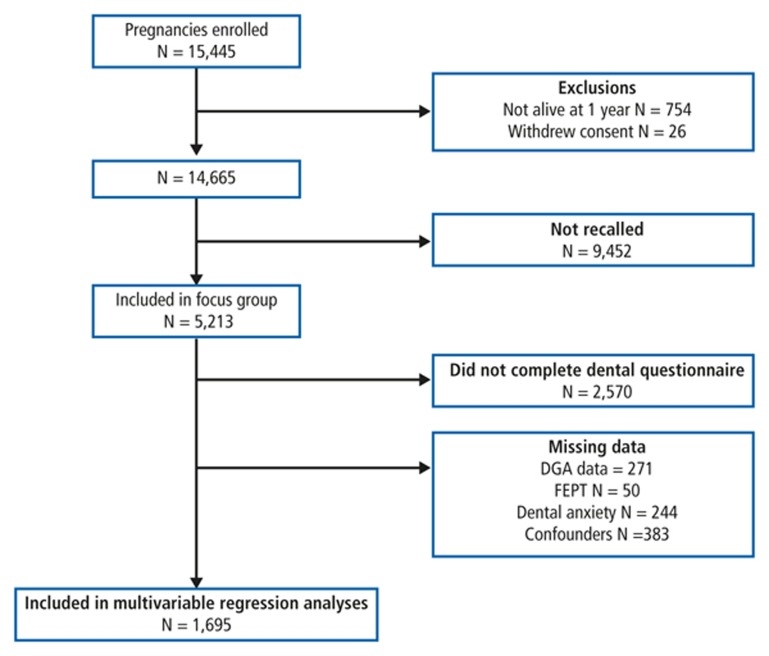
Flow of participants through study

A subset of 5,214 participants participated in a 'transitioning to adulthood' focus group at age 17, of which 2,643 answered a questionnaire about dental health. Exposure and confounder data were recorded via parent-report and participant self-report questionnaires at several time points. In total, complete exposure, outcome and confounder data were available for 1,695 participants. The flow of participants through the study and details of missing data are given in more detail in Figure 1.

### GDA status at age 7

Whether a child had received a DGA by age 7 years was identified in any parental-report questionnaire (child ages: 3, 4, 5 and 6 years) or a parent and child self-report questionnaire at age 7 years. Participants with no answer to this question in any questionnaire were excluded from analyses. The 7-year age cut-off was selected as it provides a larger sample size than earlier data collections and although slightly later than the eruption of the first permanent teeth, the majority of teeth extracted during a DGA will have been primary teeth.

### Outcome variables at age 17

Self-report questionnaires at 17 years were completed by participants attending the focus group. Questions identified how many of the participant's teeth had fillings and how many teeth had been extracted because of tooth decay. To remove the chance of a participant counting teeth extracted before and during a DGA we only included teeth extracted within the last two years. These two measures were combined into a count of filled or extracted permanent teeth (FEPT). Untreated caries was not identified.

Dental anxiety at age 17 years was identified using a Corah Dental Anxiety Scale.^19^ Where participants had missing data from more than one of the four questions their score was set to missing. We used a score of 13 or higher to identify a participant with dental anxiety as suggested by Corah *et al*.^20^

### Explanatory variables

A number of factors are likely to be related to both whether a participant has had a DGA and dental health outcomes. Sex, age at outcome measure, mother's highest educational level as a proxy for SES and dental health at age 7 were used to correct regression analyses.

Dental health at age 7 was identified from questionnaires completed by the child participant with assistance from their parent. Children were advised to use a mirror and count fillings and teeth with holes. These were combined into a count of decayed and filled teeth.

Participants' opinions of DGA was assessed by questionnaire at age 7 years and dental attendance pattern assessed by questionnaire at age 17 years; in order to improve sample size these were analysed even in those where other data were missing.

Details of questions asked at each questionnaire are provided in the supplementary material.

### Statistical analysis

Mean and standard deviation (SD) were presented for continuous variables and numbers and percentages for categorical variables, stratified by DGA status. Ttests or chi-squared tests were used to test for differences between DGA groups.

Median and interquartile range (IQR) FEPT is presented for each DGA group across SES strata to investigate the association between these two variables.

Multivariable zero-inflated negative binomial (ZINB) regression was used to test the association between DGA and FEPT at age 17. This method takes into account the non-normal distribution of FEPT with a high numbers of zero counts.^21^ Whether a child had no self-reported caries at age 7 was used to predict zero inflation. In these analyses we present four models: 1 – minimally adjusted for age and sex; 2 – adjusted for age, sex and SES; 3 – adjusted for age, sex, SES and dental anxiety at 17 years; 4 - adjusted for age, sex, SES, dental anxiety at age 17 years and dental health at age 7 years.

Logistic regression was used to investigate the association between dental anxiety and DGA as well as dental attendance patterns.

## Results

The number of participants with no missing data was 1,695; 128 (7.6%) of these received a DGA. Mean age of participants at follow up was 17.7 years and approximately 60% of participants were female, with no age or sex differences between DGA groups. Mothers of participants who had received DGA had lower educational levels than those who had not received DGA. Baseline dental health (age 7 years) was worse in those who had received a DGA. Those in the DGA group were more likely to be classified as dentally anxious than those who had not had a GA (Table 1).

### Dental health at age 17

The overall proportion of the sample with FEPT greater than zero was 0.54 (95% CI: 0.52, 0.56), in the non-DGA group this was 0.52 (95% CI: 0.50, 0.55) and in DGA group this was 0.73 (95% CI: 0.64, 0.80).

**Figure 2:**
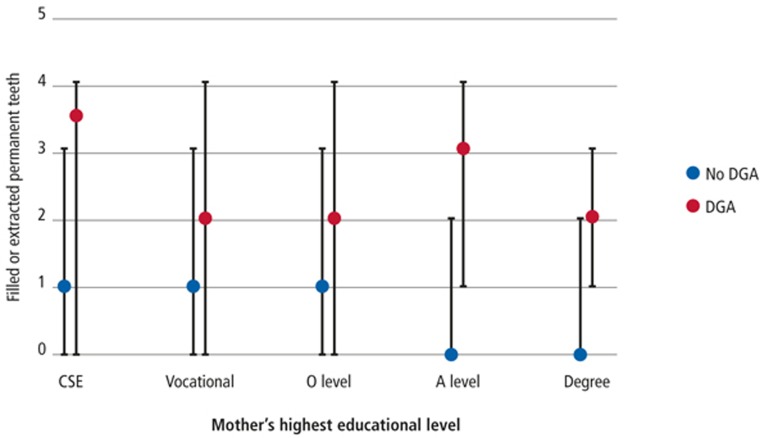
Comparison of median and interquartile range of filled extracted permanent teeth in those with and without history of dental general anaesthesia across socioeconomic strata.

Participants with a history of DGA had greater FEPT than their peers. The trend across SES strata is similar in both groups; with median FEPT in the DGA group approximately two units greater than that of the non-DGA group (Fig. 2).

In analyses adjusted only for age and sex, those who had received a DGA had 0.52 (95% CI: 0.39, 0.72; P <0.001) more units of FEPT at age 17 years than those who did not have a DGA. After adjusting for SES, dental anxiety at age 17 years and dental health at age 7 years, DGA still accounted for 0.36 (95% CI: 0.17, 0.55; P <0.001) more units of FEPT at age 17 years.

**Figure 3:**
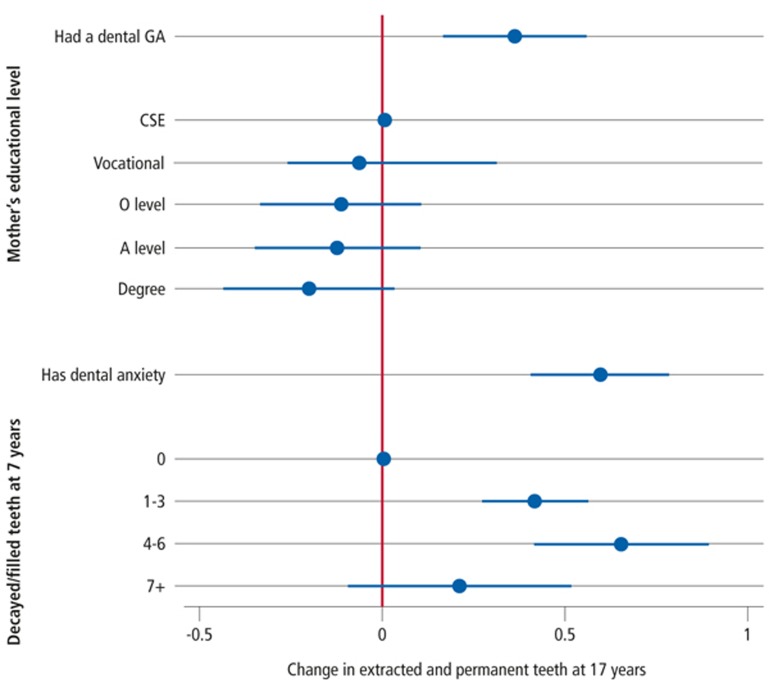
Effect of dental general anaesthesia, mothers educational level, dental anxiety and childhood caries on the number of filled and extracted permanent teeth at age 17 after correcting for the remaining explanatory variables..

Compared to the reference group (no decayed or filled teeth at age 7) and with all other variables equal, having 1–3 decayed or filled teeth at 7 years accounted for 0.41 (95% CI: 0.27, 0.56; P <0.001) more units of FEPT at age 17 years. History of a DGA accounted for 0.36 (95% CI: 0.17, 0.55; P <0.001) more FEPT units compared to children who have not undergone DGA. Dental anxiety was associated with 0.59 (95% CI: 0.40, 0.78; P <0.001) more units of FEPT at age 17 years when compared to those not dentally anxious. Although the trend suggests that higher SES is protective, when other factors are taken into account, it appeared to have only a small effect on dental health at age 17 (Fig. 3).

### Dental anxiety at age 17

The proportion of participants who were dentally anxious was 0.07 (95% CI: 0.06, 0.09). Dental anxiety was higher in the DGA group (0.20 [95% CI: 0.14, 0.28]) compared to the non-DGA group (0.07 [95% CI: 0.06, 0.08]) (P <0.001).

Participants in the DGA group had 3.62 times greater odds (95% CI: 2.22, 5.91; P <0.001) of being dentally anxious compared to those in the no DGA group. This was slightly attenuated after correcting for SES and FEPT at age 17 years (OR = 2.53 [95% CI: 1.50, 4.26; P <0.001]).

We examined the responses of children aged 7 years within ALSPAC who were asked about the experiences of undergoing DGA. Seven hundred and twenty five children responded and 290 (40.0%) said they 'hated it'. We also examined whether dental anxiety influenced patterns of dental attendance. We found evidence to suggest that dentally anxious adolescents were more likely to be symptomatic attenders, going to the dentist only when they were in pain (unadjusted OR = 3.54 [95% CI: 1.94, 6.44; *P*<0.001] N = 2,333).

## Discussion

In this population, DGA status at age 7 years was informative at identifying people on a poor oral health trajectory. Children who underwent DGA for disease in the primary dentition reported higher levels of treated caries in the permanent dentition by age 17 years.

In multivariable regression analyses, which assessed a number of explanatory variables simultaneously, extent of caries at age 7 years emerged as the single largest risk factor for the extent of treated caries in permanent teeth by age 17 years. This supports existing evidence that disease in primary dentition is a good predictor of caries prevalence in adolescence.^22^ Despite an attenuated effect size, DGA status continued to explain a difference in FEPT nearly as large as that accounted for by 1–3 carious teeth at age 7. This demonstrates that DGA status can be informative when assigning patients to caries risk categories. This is true even when considered independently of preconceived risk factors, but also implies that the apparent detrimental effect of DGA on dental health in adolescence is not fully explained by DGA status proxying preconceived risk factors.

The multivariable regression analyses highlighted a relationship between dental anxiety at age 17 years and treated caries at age 17 years, with higher levels of treated caries in people who had dental anxiety. There is scope for bidirectional causality in this relationship. People who have undergone more invasive procedures as a consequence of disease may have more negative experiences of dental disease or treatment and develop dental anxiety. Alternatively, people who are anxious may avoid attending a dentist for routine care, leading to higher levels of disease. Existing evidence supports the latter argument, suggesting both that individuals with anxiety avoid dental attendance, and that anxiety is a risk factor for dental caries in adolescents.^23^,^24^

As well as the relationship between dental anxiety at age 17 and treated caries at age 17, this analysis highlighted a relationship between DGA status and dental anxiety at age 17.

People who received a DGA were over 2.5 times more likely to be anxious at age 17 years. Once again, there is scope for bidirectional causality in this relationship. Though beyond the scope of this work, further efforts are needed to untangle the relationship between DGA and anxiety which are likely to interact with each other in a cyclical manner. Some children may have undergone DGA as a consequence of anxiety meaning they were unable to receive treatment under other methods of pain or anxiety control. Alternatively, negative events at the time of DGA could be a risk factor for future anxiety.

The value of DGA in managing dental caries is well established. There is a positive impact on oral health quality of life in the short term following treatment.^25^ At the present time there is no evidence to show that this improvement is sustained beyond nine months after DGA. Alongside the reports of positive impacts of DGA there are reports of negative impacts on children and their families.^26^,^27^,^28^

We have not tested a causal effect, however we speculate that DGA may influence dental anxiety, which is known to lead to altered dental attendance and in turn could lead to higher levels of disease over the longer term. This mechanism could explain why DGA status is strongly associated with dental health in adolescence, even after taking into account dental disease in childhood, but further investigation is required.

DGA status could be used to direct prevention and resources to individuals at greatest need. An ideal opportunity for this is concurrent with the timing of the DGA. In the UK, DGA is now performed exclusively in a hospital setting, usually following referral from a general dental practitioner and assessment by a specialist paediatric dentist. This means that, before treatment, children and their families may have contact with dental professionals and specialist services on multiple occasions. This is a rare situation in which a known high-risk group seeks healthcare services, providing an important opportunity for future prevention through oral health education by dental professionals.

FEPT is not a clinical measure and does not lend itself to direct comparison with other measures. However, in terms of caries prevalence the 2013 child dental health survey reported 44% of 15-year-olds in England had obvious caries experience in England, compared with 32% of 12-year-olds.^1^ We report that 54% of participants have FEPT >0, which seems broadly in line with expectations when taking into account the older age of our participants.

The proportion of participants with dental anxiety in this study (defined as Corah's anxiety scale > = 13) was 8% which is not incompatible with 19% in females aged 16–34 and 8% in males aged 16–34 in the UK (defined as MDAS > = 19).^(29)^

The index of treated caries presented in this study is derived from questionnaire data and is not directly comparable to a clinical index. The method used to calculate FEPT does not capture untreated caries or caries treated by extraction more than two years before age 17 years. In addition, self-reported caries measures will under-report dental caries compared to an objective clinical examination. Previous research within ALSPAC comparing clinical and self-reported caries measures corroborates this under-reporting.^30^ The results from this study should therefore be considered as an underestimation of the true situation and potentially confounded by directional bias in dental under-reporting.

There is substantial attrition from the ALSPAC study, that has resulted in selective drop out of participants from lower SES groups.^31^ These groups have higher levels of dental caries, meaning that results from the remaining participants may further underestimate true caries experience in the general population.

The participants in this study underwent DGA in the late 1990s. Therefore, our findings may reflect historical preventive practice that may have changed since. Additionally, the participants in this study were recruited from a single geographical area in the former county of Avon and so our results may not be generalisable to other populations.

The scope of this study is limited by available prospectively obtained data. Although we highlight longer-term outcomes for young people who received DGA in childhood, we can neither provide a detailed oral health trajectory through the mixed dentition nor establish the effects of oral health interventions.

## Conclusions

This study highlights that children who undergo DGA are a high-risk group for poor oral health and dental anxiety as they get older. DGA status predicts burden of treated caries ten or more years after the procedure itself independently of past caries experience. DGA status is associated with greatly increased odds of dental anxiety in adolescence. Regardless of the underlying mechanism, DGA status could be used in clinical practice or risk assessment models in identifying people at high risk of further disease or anxiety.

This study also highlights important gaps in our knowledge about the longer term health of children who undergo DGA, which this study can only go part way to addressing. It is possible that negative events at the time of DGA could have downstream influence, leading to worse dental anxiety and dental health ten or more years later. With ever increasing numbers of children undergoing DGA in the UK, it is important to establish whether this effect is seen in other studies with regular, prospectively obtained data.

## Figures and Tables

**Table 1:** Baseline characteristics by dental general anaesthetic (DGA) status

	All	DGA	No DGA	P-value
Mean (SD) Age (years)	17.7 (0.4)	17.7 (0.4)	17.7 (0.4)	0.317
N (%) Female	1,021 (60.2)	81 (63.3)	940 (60.0)	0.464
N (%) Mothers highest educational level				<0.001
CSE	136 (8.0)	24 (18.8)	112 (7.2)	
Vocational	114 (6.7)	13 (10.2)	101 (6.5)	
O level	545 (32.2)	46 (35.9)	499 (31.8)	
A level	497 (29.3)	26 (20.3)	471 (30.0)	
Degree	403 (23.8)	19 (14.8)	384 (24.5)	
Decayed/filled teeth at 7 years				<0.001
0	1,116 (65.8)	54 (42.2)	1,062 (67.7)	
1 - 3	439 (25.9)	54 (42.2)	385 (24.6)	
4 - 6	81 (4.8)	10 (7.8)	71 (4.5)	
7+	59 (3.5)	10 (7.8)	49 (3.1)	
N (%) Dentally anxious	130 (7.7)	26 (20.3)	104 (6.6)	<0.001
N(%)	1,695 (100)	128 (7.6)	1,567 (92.4)	
CSE – certificate of secondary education, DGA – Dental general anaesthetic, N – number, SD – standard deviation				

## Notes

**Refereed Paper**
